# Verhütung in der deutschsprachigen Wikipedia: Eine Inhalts- und Qualitätsanalyse

**DOI:** 10.1007/s00103-022-03537-8

**Published:** 2022-04-26

**Authors:** Nicola Döring, Stephan Lehmann, Claudia Schumann-Doermer

**Affiliations:** 1 Institut für Medien und Kommunikationswissenschaft (IfMK), Technische Universität Ilmenau, Ehrenbergstraße 29, 98693 Ilmenau, Deutschland; 2 Deutsche Gesellschaft für psychosomatische Frauenheilkunde und Geburtshilfe (DGPFG), Dresden, Deutschland

**Keywords:** Gesundheitsinformationen, Verhütungsinformationen, Informationsqualität, Internet, DISCERN-Index, Health information, Contraceptive information, Information quality, Internet, DISCERN index

## Abstract

**Hintergrund:**

Jugendliche und Erwachsene beziehen Informationen über Verhütung heute oft per Internet, vor allem über die Online-Enzyklopädie Wikipedia, da Google-Suchen meist Wikipedia-Einträge als Toptreffer liefern.

**Ziel der Arbeit:**

Vor diesem Hintergrund ist es Ziel des vorliegenden Beitrags, erstmals Inhalte und Qualität von Wikipedia-Artikeln über Verhütungsmethoden systematisch zu analysieren. Geprüft werden dabei 5 zentrale Qualitätsdimensionen: die Ausprägung der Korrektheit (Forschungsfrage F1), der Vollständigkeit (F2), der Neutralität (F3), der Verständlichkeit (F4) und der Aktualität (F5) der Verhütungsinformationen sowie auf dieser Basis auch ihre Gesamtqualität (F6).

**Material und Methoden:**

Es wurde eine Stichprobe aller deutschsprachigen Wikipedia-Artikel zu allen Verhütungsmethoden gebildet (*N* = 25). Diese Artikel wurden mittels eines auf der Basis des Forschungsstandes entwickelten und reliabilitätsgeprüften Codebuchs von 3 unabhängigen, geschulten Codierenden analysiert. Die Datenanalyse erfolgte mit SPSS. Die Studie ist präregistriert und alle Daten, Materialien und Analyseskripte sind öffentlich verfügbar.

**Ergebnisse:**

Es zeigte sich, dass die 25 Wikipedia-Artikel zu Verhütungsmethoden in ihrer inhaltlichen Qualität stark variierten. Während sie hinsichtlich Korrektheit (F1) und Neutralität (F3) im Mittel gute Qualität aufwiesen, erreichten sie hinsichtlich Vollständigkeit (F2), Verständlichkeit (F4) und Aktualität (F5) nur mittelmäßige Werte, woraus sich dann auch eine moderate Gesamtqualität ergab (F6).

**Diskussion:**

Weitere Forschung sowie Praxismaßnahmen sind notwendig, um die Qualität von Verhütungsinformationen in der Wikipedia und in anderen sozialen Medien noch besser einschätzen und zielgerichteter verbessern zu können.

## Abstract (en)

**Background:**

Adolescents and adults today often obtain information about contraception from the Internet, especially from the online encyclopedia Wikipedia. This is because Google searches usually return Wikipedia entries as top hits.

**Research aim:**

Against this background, the aim of the current study is to systematically analyze for the first time the content and quality of Wikipedia articles on contraceptive methods. Five central quality dimensions are examined: the degree of correctness (research question RQ1), completeness (RQ2), neutrality (RQ3), comprehensibility (RQ4), and currency (RQ5) of the contraceptive information – and on this basis the overall quality of the articles (RQ6).

**Materials and methods:**

A sample of all German-language Wikipedia articles on all contraceptive methods was formed (*N* = 25). These articles were analyzed by three independent, trained coders using a codebook that was developed based on the current state of the research and tested for reliability. Data analysis was performed using SPSS. The study is preregistered and all data, materials, and analysis scripts are publicly available.

**Results:**

The 25 Wikipedia articles on contraceptive methods were found to vary widely in content quality. While they showed good quality on average in terms of correctness (RQ1) and neutrality (RQ3), they scored mediocre in terms of completeness (RQ2), comprehensibility (RQ4), and currency (RQ5), resulting in moderate overall quality (RQ6).

**Discussion:**

More research as well as practice measures are needed to further assess and improve the quality of contraceptive information on Wikipedia and in other social media in a more targeted way.

## Hintergrund

*Empfängnis- bzw. Zeugungsverhütung* (kurz: Verhütung, engl. „contraception“) meint alle Verhaltensweisen, Mittel und Methoden, die absichtlich angewendet werden, um trotz Geschlechtsverkehr eine Empfängnis bzw. Zeugung zu verhindern [1]. Das Spektrum der Verhütungsmethoden umfasst traditionelle Verhaltensweisen (z. B. Coitus interruptus) sowie moderne medizinische Mittel (z. B. Antibabypille; [2]), kurzfristig (z. B. Kondom) sowie langfristig (z. B. Spirale) wirkende Methoden [3], hormonelle (z. B. Hormonspirale) sowie nichthormonelle (z. B. Kupferspirale) Verfahren [4]. Die einzelnen Verhütungsmethoden^1^ unterscheiden sich in ihrer Sicherheit sowie in diversen weiteren Merkmalen wie etwa Wirkungsmechanismus, Anwendung und Kosten.

Es wird heute als *sexuelles und reproduktives Menschenrecht* angesehen, allen Menschen die Möglichkeit zu geben, durch Informationen über moderne Verhütungsmethoden und den Zugang zu ihnen selbst über die eigene Fortpflanzung bestimmen zu können [5] und somit ungeplante und ungewollte Schwangerschaften und deren Negativfolgen zu verhindern [6]. Beim Zugriff auf Verhütungsinformationen spielt das Internet eine zentrale Rolle: So geben in Deutschland Frauen im Alter zwischen 18 und 49 Jahren die gynäkologische Praxis (80 %) und das Internet (29 %) als ihre beiden wichtigsten Informationsquellen zu Verhütungsmethoden an, Männer im selben Alter nennen das Internet (40 %) sowie Familie und Freunde (40 % [7]), Jugendliche nennen Schulunterricht (69 %), Gespräche (68 %) und das Internet (59 % [8]).

Empirische Studien zeigen, dass sich unter den ersten Google-Treffern *Wikipedia-Einträge* befinden, wenn „Antibabypille“ oder „Kondom“ in die Suchmaske eingegeben wurde [9, 10]. Die große Reichweite der Wikipedia als Informationsquelle zu Verhütungsmethoden lässt sich auch daran erkennen, dass beispielsweise der deutschsprachige Wikipedia-Eintrag zum Kondom^2^ im Jahr 2020 über 225.000-mal abgerufen wurde, also rund 620-mal pro Tag. Diese Reichweite übertrifft deutlich jene von offiziellen Aufklärungsseiten^3^. So verzeichnete das Portal der Bundeszentrale für gesundheitliche Aufklärung (BZgA) www.familienplanung.de im Jahr 2020 beispielsweise rund 50.000 Aufrufe^4^ seines Beitrags über das Kondom^5^, das entspricht knapp 150 Aufrufen pro Tag.

Angesichts der großen Reichweite von Verhütungsinformationen in der Wikipedia und verbreiteten Zweifeln an ihrer Zuverlässigkeit [11, 12] stellt sich die Frage, wie die verschiedenen Verhütungsmethoden in der Wikipedia dargestellt sind und welche Inhaltsqualität diese Wikipedia-Einträge haben. Der vorliegende Forschungsbeitrag geht diesen Fragen erstmals systematisch nach. Dazu werden zunächst Forschungsstand, Forschungsziel und Forschungsmethoden erläutert, bevor die Ergebnisse präsentiert und diskutiert werden.

### Forschungsstand

Das gemeinnützige und ehrenamtlich getragene Wikipedia-Projekt wurde 2001 ins Leben gerufen, um eine freie, kollektiv erstellte Internet-Enzyklopädie zu entwickeln. Das Projekt ist insofern als Erfolg anzusehen, als die Wikipedia heute sehr viele verschiedene Themenbereiche abdeckt und zu den populärsten Internetadressen gehört: Weltweit rangiert die Wikipedia auf Platz 13, in Deutschland auf Platz 6 der meistbesuchten Websites [13]. Es ist der Selbstanspruch der Wikipedia als Online-Enzyklopädie nur Einträge vorzuhalten, die Relevanz- und Qualitätskriterien genügen. Sichergestellt wird dies durch ein komplexes Regelwerk und Selbstkontrollsystem [14].

Wie gut die internen Steuerungsmechanismen funktionieren und welche Inhaltsqualität Wikipedia-Einträge am Ende haben, wird seit rund 2 Dekaden untersucht (für Forschungsübersichten siehe z. B. [15] oder [16]). Ein inhaltlicher Schwerpunkt liegt dabei auf *medizinischen Informationen in der Wikipedia*, da die Wikipedia inzwischen als die weltweit meistgenutzte Online-Quelle für medizinisches Wissen gilt [10, 17]. Einige Studien bescheinigen der gemeinschaftlich von einer Vielzahl von Menschen erstellten (Crowd-basierten) Wikipedia eine Inhaltsqualität, die mit derjenigen von Quellen vergleichbar ist, die von Fachleuten erstellt werden, – etwa herkömmlichen Enzyklopädien [18, 19], Sachbüchern [20] und Krankenkassenwebsites [21]. Gleichzeitig weisen die vorliegenden Studienergebnisse auf deutliche Qualitätsunterschiede innerhalb der Wikipedia hin: So werden Wikipedia-Einträge zu Themen von großer öffentlicher Bedeutung häufiger gelesen und überarbeitet, was zu besserer Qualität führt im Vergleich zu Einträgen über Nischenthemen [22, 23].

Wir konnten keine einzige Studie identifizieren, die speziell Wikipedia-Einträge zur Verhütung betrachtet. Dafür liegen 10 Studien vor, die Verhütungsinformationen auf Social-Media-Plattformen wie Youtube, TikTok, Instagram, Reddit und Twitter analysieren [11, 24–31]. Diese Einzelstudien weisen ebenso wie breitere Übersichtsarbeiten zu sexuellen und reproduktiven Online-Gesundheitsinformationen [12, 32, 33] auf unterschiedliche Qualitätsmängel hin.

### Forschungsziel

Angesichts der Tatsache, dass die Verhütungsinformationen in der Wikipedia sehr reichweitenstark sind und bislang noch nie systematisch analysiert wurden, verfolgt die vorliegende Studie das Ziel, diese Forschungslücke zu schließen. Untersucht werden alle deutschsprachigen Wikipedia-Beiträge zu allen verfügbaren Verhütungsmethoden.

Dafür wird ein Qualitätsmodell herangezogen, das die Verhütungsinformationen in der Wikipedia anhand von fünf zentralen Qualitätsdimensionen bewertet: 1. Korrektheit (Evidenzbasierung), 2. Vollständigkeit, 3. Neutralität, 4. Verständlichkeit und 5. Aktualität der Informationen. Das Modell stützt sich auf die bisherige Forschung zu Inhalten und Qualität von Gesundheitsinformationen im Internet und in der Wikipedia [10]. Die fünf zentralen Qualitätsdimensionen sind Bestandteil des führenden Instruments zur Messung der Qualität von Online-Gesundheitsinformationen, dem *Modified-DISCERN-Index*^6^ [35, 36], der unter anderem in der Wikipedia-Forschung [35–40] eingesetzt wird. Die fünf Qualitätskriterien sind zudem enthalten in der Leitlinie evidenzbasierter Gesundheitsinformation [41]. Aus dem fünfdimensionalen Qualitätsmodell abgeleitet ergeben sich bezüglich der Einträge zu Verhütungsmethoden in der deutschsprachigen Wikipedia fünf Forschungsfragen:

#### F1.

Wie korrekt (evidenzbasiert) sind die Einträge?

#### F2.

Wie vollständig sind die Einträge?

#### F3.

Wie neutral sind die Einträge?

#### F4.

Wie verständlich sind die Einträge?

#### F5.

Wie aktuell sind die Einträge?

Auf der Basis der Betrachtung der einzelnen Dimensionen ergibt sich eine sechste Frage zur Gesamtqualität:

#### F6.

Wie ist die Gesamtqualität der Einträge einzuschätzen?

## Methoden

### Untersuchungsdesign

Die vorliegende Inhalts- und Qualitätsanalyse ist als quantitative Querschnittstudie zu kennzeichnen, wobei das Untersuchungsmaterial aus öffentlich frei zugänglichen Wikipedia-Einträgen besteht, die nach aktuellem Verständnis der Online-Forschungsethik für wissenschaftliche Untersuchungen frei zur Verfügung stehen [42]. Die Studie ist präregistriert und folgt der Open-Science-Bewegung, das heißt, die Präregistrierung, das Messinstrument, der Datensatz und das Auswertungsskript sind auf dem Server der Open-Science-Foundation öffentlich frei zugänglich hinterlegt (https://osf.io/rebqh/).

### Stichprobe

Die Stichprobe umfasst alle *N* = 25 deutschsprachigen Wikipedia-Artikel (synonym: Wikipedia-Beiträge/-Einträge), die jeweils einer distinkten Verhütungsmethode gewidmet sind (Tab. 1). Da die Wikipedia als offene Online-Enzyklopädie dynamisch ist und Beiträge jederzeit verändert werden können, wurden die zu untersuchenden Wikipedia-Beiträge an einem Stichtag (28.07.2021) in Form von PDF-Dateien archiviert. Damit war sichergestellt, dass alle Analysen sich stets auf denselben Stand eines Wikipedia-Beitrags beziehen.

**Table 1:** 

Verhütungsmethode	Erstellungsdatum	Zeichen gesamt	Autor*innen	Bearbeitungen gesamt	Bearbeitungen pro Monat
**1. Antibabypille**	19.07.2003	**28.439**	**247**	**1402**	**6,5**
2. Coitus interruptus	21.07.2003	4827	54	442	2,1
3. Diaphragma	05.08.2003	3532	46	498	2,3
4. Dreimonatsspritze	17.05.2006	3149	32	321	1,8
5. Etonogestrel-Implantat	12.03.2004	6978	54	249	1,3
6. Femidom	10.07.2003	1787	66	304	1,4
7. Hormonpflaster	12.03.2004	2110	35	208	1,0
8. Hormonspirale	11.10.2004	6666	55	336	1,7
9. Intrauterinpessar	11.03.2004	11.051	85	406	2,0
10. Knaus-Ogino-Methode	22.12.2003	5389	47	252	1,2
**11. Kondom**	17.09.2002	**21.393**	**278**	**1499**	**6,6**
12. Kupferkette	29.01.2009	3547	23	77	0,6
13. LAM-Methode	02.08.2006	4594	29	78	0,5
14. Minipille	22.12.2003	2788	32	167	0,8
15. Pille danach	18.09.2002	19.974	150	847	3,9
16. Pille für den Mann	29.07.2005	3920	49	167	0,8
17. Portiokappe	05.12.2003	2988	41	135	0,7
18. Spermizid	05.12.2003	1696	24	88	0,4
19. Sterilisation der Frau^**a**^	17.10.2002	8708	84	421	1,9
20. Sterilisation des Mannes	19.07.2003	9751	79	403	1,9
**21. Symptothermale Methode**	**21.12.2003**	**23.934**	**79**	**507**	**2,4**
22. Temperaturmethode	21.12.2003	2552	35	257	1,2
23. Verhütungsring	25.06.2004	6617	72	491	2,4
24. Verhütungsschwamm	07.04.2004	4965	53	303	1,5
25. Zykluscomputer	29.11.2004	2783	43	211	1,1
**Mittelwert M (insgesamt)**	**2004** (SD = 1,51)	**7765,52** (SD = 7349,88)	**71,38** (SD = 63,58)	**402,76** (SD = 359,04)	**1,92** (SD = 1,59)

Die 3 längsten Artikel (> 20.000 Zeichen) sind durch Fettdruck hervorgehoben

*LAM-Methode* Laktationsamenorrhö-Methode, Methode der natürlichen Empfängnisverhütung durch Stillen

^a^Zu allen Verhütungsmethoden liegt jeweils ein eigener Wikipedia-Artikel vor, mit Ausnahme der Sterilisation der Frau, die als Abschnitt im Beitrag „Sterilisation“ behandelt wird

### Instrument

Als Messinstrument wurde ein Codebuch (Tab. 2) mit Checklisten erstellt. Das Codebuch wurde deduktiv anhand des aus der Literatur abgeleiteten Qualitätsmodells entwickelt und induktiv anhand einer ersten Sichtung von Wikipedia-Einträgen ergänzt. Unser Messinstrument basiert auf dem Modified-DISCERN-Index [36] und beinhaltet zudem den Lesbarkeitsindex Flesh Reading Ease für deutschsprachige Texte nach Amstad [43]. Weiterhin wurde aktuelle gynäkologische Fachliteratur hinzugezogen [44–46] und die Expertise einer auf Verhütung spezialisierten gynäkologischen Fachärztin (Autorin 3) in die Entwicklung und Validierung des Codebuchs einbezogen. Wir haben methodisch sehr großen Wert auf eine transparente Qualitätsbeurteilung gelegt, das heißt, im Codebuch ist genau hinterlegt, welche zentralen Informationen beispielsweise über die Antibabypille laut aktueller gynäkologischer Fachliteratur verfügbar sind und somit prinzipiell in der Wikipedia genannt werden können, sodass der Grad der (Un)Vollständigkeit objektiv daraus abzuleiten ist, wie viele dieser Informationen auszählbar im Wikipedia-Beitrag auftauchen. Damit basieren die Qualitätsdaten unserer Studie nicht auf rein subjektiven Eindrücken^7^, sondern auf detailliert anhand des aktuellen Forschungsstandes zu Verhütungsmethoden ausgearbeiteten Checklisten, die als Teil des Codebuchs hinterlegt sind. Für jede der 25 Verhütungsmethoden wurde eine eigene Checkliste entwickelt, bei der ihre jeweiligen spezifischen Merkmale berücksichtigt werden.

**Table 2:** 

Zentrale Qualitätsdimensionen für Gesundheitsinformationen	10 Items des Modified-DISCERN-Index	Anpassung und Konkretisierung der Modified-DISCERN-Items für die Analyse von Wikipedia-Artikeln zu Verhütungsmethoden Zuweisung der Scores für die Modified-DISCERN-Items (jeweils Ratingskala 1–5)	Messung der Merkmale des Wikipedia-Artikels
**1. Korrektheit (Evidenzbasierung)**	1. Sind die Ziele der Darstellung zu Beginn des Artikels klar formuliert?^a^	1 = Verhütungsmethode ist nicht klar/korrekt benannt 3 = Verhütungsmethode ist klar/korrekt benannt 5 = Verhütungsmethode ist klar/korrekt benannt mit Synonymen	Der Wikipedia-Artikel wird direkt eingeschätzt bezüglich Zielbenennung
	2. Sind die in dem Artikel enthaltenen Informationen wissenschaftlich korrekt und stimmen sie mit den aktuell gültigen Quellen und Lehrbüchern überein?	1 = weniger als 70 % der gegebenen Informationen sind korrekt 2 = 70–79 % der gegebenen Informationen sind korrekt 3 = 80–89 % der gegebenen Informationen sind korrekt 4 = 90–99 % der gegebenen Informationen sind korrekt 5 = 100 % der gegebenen Informationen sind korrekt	Der Anteil der korrekten Informationen (Unterthemen) an allen Informationen über die jeweilige Verhütungsmethode im Wikipedia-Artikel wird anhand der Checkliste für die jeweilige Methode ermittelt
	3. Ist klar, welche Informationsquellen bei der Erstellung der Veröffentlichung verwendet wurden (Referenzen, Links zu professionellen Websites)?	1 = weniger als 25 % der Informationen mit Quellen belegt 2 = 25–49 % der Informationen mit Quellen belegt 3 = 50–74 % der Informationen mit Quellen belegt 4 = 75–99 % des Textes mit Quellen belegt 5 = Informationen durchgängig (100 %) mit Quellen belegt	Der Anteil der mit Quellen hinterlegten Textabschnitte an allen Textabschnitten im Wikipedia-Artikel wird ermittelt
**2. Vollständigkeit**	4. Enthält der Artikel die erforderlichen Hauptthemen und Schlüsselkonzepte im Zusammenhang mit dem Thema?	1 = 3 oder weniger Hauptthemen werden behandelt 2 = 4 Hauptthemen werden behandelt 3 = 5 Hauptthemen werden behandelt 4 = 6 Hauptthemen werden behandelt 5 = alle 7 Haupthemen werden behandelt	Ausgezählt wird, wie viele der 7 relevanten Hauptthemen bezüglich jeder Verhütungsmethode im Wikipedia-Artikel behandelt werden (1. Wirkungsmechanismus, 2. Sicherheit, 3. Anwendung, 4. Vorteile, 5. Nachteile, 6. Kosten, 7. Gegenanzeige)
	5. Sind die Unterthemen des Artikels vollständig und bedürfen keiner weiteren Maßnahmen?	1 = weniger als 55 % der Unterthemen werden behandelt 2 = 55–69 % der Unterthemen werden behandelt 3 = 70–84 % der Unterthemen werden behandelt 4 = 85–99 % der Unterthemen werden behandelt 5 = alle Unterthemen werden behandelt	Dokumentiert wird der prozentuale Anteil der im Wikipedia-Artikel vorhandenen Unterthemen an der Anzahl aller möglichen Unterthemen einer Verhütungsmethode mittels dazugehöriger Checkliste (z. B. zählen beim Hauptthema „Sicherheit“ die Nennung des Pearl-Index bei üblicher versus perfekte Nutzung als Unterthemen)
**3. Neutralität**	6. Ist der Artikel neutral und basiert er nicht auf persönlichen Ansichten?	1 = sehr geringe Neutralität 2 = geringe Neutralität 3 = mittlere Neutralität 4 = hohe Neutralität 5 = sehr hohe Neutralität	Der Wikipedia-Artikel wird direkt eingeschätzt bezüglich Neutralität im Sinne einer sachlichen, nicht polarisierenden oder persönlich wertenden Darstellung
	7. Ist der Artikel ausgewogen und unbeeinflusst geschrieben?	1 = sehr geringe Ausgewogenheit 2 = geringe Ausgewogenheit 3 = mittlere Ausgewogenheit 4 = hohe Ausgewogenheit 5 = sehr hohe Ausgewogenheit	Der Wikipedia-Artikel wird direkt eingeschätzt bezüglich Ausgewogenheit im Sinne einer angemessenen Darstellung der wissenschaftlich belegten Vor- und Nachteile einer Verhütungsmethode
**4. Verständlichkeit (durch Visualisierungen)**^**b**^	8. Unterstützen die im Artikel enthaltenen Bilder, Abbildungen und Tabellen die gegebenen Informationen und fördern das Verständnis der angesprochenen Punkte?	1 = keine Visualisierung vorhanden 2 = eine oder mehrere Visualisierungen vorhanden, aber nicht hilfreich 3 = eine hilfreiche Visualisierung vorhanden oder mehrere Visualisierungen vom Produkt, die sich ähneln 4 = mehr als eine hilfreiche Visualisierung vorhanden, aber damit werden nicht alle gegebenen Informationen unterstützt (z. B. Kondom und Kondompackung, aber keine Visualisierung der Anwendung) 5 = mindestens 2 hilfreiche Visualisierungen unterschiedlicher Arten, die alle gegebenen Informationen unterstützen, z. B. vom Produkt und von der Anwendung/Verwendung	Dokumentiert werden Anzahl und Art der Visualisierungen, auf dieser Basis werden die Verständlichkeitsratings vergeben
**5. Aktualität**	9. Wurde der Artikel regelmäßig aktualisiert und geändert?	1 = Der Artikel wurde innerhalb der letzten 12 Monate nicht bearbeitet 2 = Der Artikel wurde zwar innerhalb der letzten 12 Monate bearbeitet (z. B. „kleine Bearbeitungen“), aber inhaltlich nicht verändert, zudem existieren tote Links 3 = Der Artikel wurde innerhalb der letzten 12 Monate inhaltlich nicht verändert, aber es erfolgten andere Bearbeitungen (z. B. „kleine Bearbeitungen“) und es existieren keine toten Links 4 = Der Artikel wurde mindestens einmal innerhalb der letzten 12 Monate inhaltlich verändert, aber es existieren tote Links 5 = Der Artikel wurde mindestens einmal innerhalb der letzten 12 Monate inhaltlich verändert und es existieren keine toten Links	Dokumentiert werden redaktionelle und inhaltliche Bearbeitungen der letzten 12 Monate sowie die Anzahl der toten Links, auf dieser Basis werden die Aktualitätsratings vergeben
**6. Subjektive Gesamtbewertung**	10. Wie bewerten Sie den Artikel insgesamt als Informationsquelle für Patienten?	1 = sehr geringe Qualität 2 = geringe Qualität 3 = mittlere Qualität 4 = hohe Qualität 5 = sehr hohe Qualität	Der Wikipedia-Artikel wird direkt subjektiv eingeschätzt bezüglich Gesamtqualität
***Modified-DISCERN-Score***	*Summe aller 10 DISCERN-Items*	*DISCERN-Score: 10 (schlechteste Qualität) bis 50 (beste Qualität)*	*Es wird die Summe der Scores der 10 einzelnen DISCERN-Items gebildet*
**Lesbarkeitsindex** gemäß Flesch-Reading-Ease-(FRE‑)Index nach Amstad (1978; [43])	*Bestimmt nach der Formel:* FRE = 180 − ASL − (58,5 $\cdot$ ASW). ASL = durchschnittliche Satzlänge (*Average Sentence Length*), ASW = durchschnittliche Silbenanzahl pro Wort (*Average Number of Syllables per Word*)	0–30 = sehr schwere Lesbarkeit (Niveau von Hochschulabsolvent*innen) 31–50 = schwere Lesbarkeit 51–60 = mittelschwere Lesbarkeit 61–70 = mittlere Lesbarkeit 71–80 = mittelleichte Lesbarkeit 81–90 = leichte Lesbarkeit 91–100 = sehr leichte Lesbarkeit (Niveau von 11-jährigen Schüler*innen)	Der Flesch-Reading-Ease-Index nach Amstad ist ein numerischer Wert zwischen 0 und 100 und zeigt auf, wie verständlich bzw. lesbar ein deutschsprachiger Text ist

Instrument induktiv und deduktiv entwickelt auf der Basis des Modified-DISCERN-Index von Azer et al. (2015; [36]). Zudem Erfassung des Lesbarkeitsindex für deutschsprachige Texte gemäß Flesch-Reading-Ease-Index von Amstad (1978; [43]), operationalisiert mithilfe des Onlinetools https://fleschindex.de/ von Peter Schöll (letzter Zugriff: 12.04.2022)

^a^Im Fall der Wikipedia-Einträge zu Verhütungsmethoden ist das Ziel jeweils, die benannte Methode darzustellen

^b^Die Verständlichkeit eines Wikipedia-Eintrags zu einer Verhütungsmethode wird gemäß Modified-DISCERN-Index nur an Visualisierungen bemessen, zusätzlich wurde daher der Lesbarkeitsindex für den Text erfasst (siehe letzte Zeile von Tab. 2)

Die Reliabilität des Codebuchs wurde mittels Inter-Codierer*innen-Übereinstimmung überprüft: 3 geschulte Personen codierten unabhängig voneinander alle 25 Wikipedia-Einträge und die Inter-Codierer*innen-Übereinstimmung wurde für jede Kategorie im Codebuch ermittelt mit dem Kappa-Koeffizienten von Fleiss für nominal- und ordinalskalierte Kategorien bzw. der Intra-Class-Correlation (ICC) für metrische Kategorien. Die Reliabilitätskoeffizienten rangierten zwischen 0,65 und 1,00 mit Mittelwerten für Fleiss’ Kappa = 0,86 und für ICC = 0,97, was auf durchgängig gute Messgenauigkeit hinweist [47].

### Datenerhebung und Datenanalyse

Die Datenerhebung erfolgte im Rahmen manueller Codierung im Sommer 2021 durch 3 geschulte Codierende. Grundlage der Codierung waren die oben genannten PDF-Dokumente der Wikipedia-Einträge, das Codebuch und die Checklisten. Die Datenanalyse erfolgte deskriptiv- und inferenzstatistisch unter Nutzung der Software SPSS (Version 26).

## Ergebnisse

### Stichprobenbeschreibung

Die 25 eingeschlossenen deutschsprachigen Wikipedia-Einträge wurden fast alle bereits vor knapp 20 Jahren – also kurz nach der Gründung der Wikipedia – angelegt (Tab. 1). Die Artikellänge betrug im Mittel rund 8000 Zeichen, das entspricht gut vier DIN-A4-Normseiten^8^. Im Durchschnitt hatten bislang jeweils rund 70 Personen an einem Artikel mitgearbeitet. Über die Zeit wurde jeder Artikel etwa 400-mal überarbeitet, was ca. 2 Überarbeitungen pro Monat entspricht. Bei diesen Überarbeitungen handelte es sich sowohl um kleinere Korrekturen (z. B. von Tippfehlern) als auch größere Veränderungen (z. B. Einfügen neuer Textabschnitte und neuer Quellen). Es zeigten sich sehr enge positive Zusammenhänge zwischen der Artikellänge und der Anzahl der beteiligten Autor*innen (r = 0,85, *p* < 0,001) sowie der Häufigkeit von Überarbeitungen (r = 0,84, *p* < 0,001; [23, 48]).

Die 3 umfangreichsten Artikel behandelten auf jeweils über 20.000 Zeichen die Antibabypille, die symptothermale Methode und das Kondom (Tab. 1). Die Länge der Artikel variierte zwischen 1696 Zeichen (< 1 Normseite: Spermizid) und 28.439 Zeichen (> 16 Normseiten: Antibabypille).

### Korrektheit der Verhütungsinformationen

Eine Grundanforderung an Informationen in der Wikipedia ist die inhaltliche Korrektheit bzw. Evidenzbasierung. Gemäß unserem Messinstrument (Tab. 2) wird inhaltliche Korrektheit daran festgemacht, dass 1) der Wikipedia-Artikel die dargestellte Verhütungsmethode korrekt (auch mit Synonymen) benennt, 2) dass die im Artikel enthaltenen Informationen über die Verhütungsmethode wissenschaftlich zutreffend sind und 3) dass für die im Wikipedia-Artikel enthaltenen Informationen entsprechende Quellennachweise angegeben sind. Die Werte dieser 3 DISCERN-Items wurden zu einem Korrektheitswert mit dem Wertebereich 1 (geringste Korrektheit) bis 5 (höchste Korrektheit) zusammengefasst.

Es zeigte sich, dass die Bewertung des Wikipedia-Eintrags zur Dreimonatsspritze hinsichtlich Korrektheit mit einem Score von 1,67 am schlechtesten ausfiel (Tab. 3). Denn zum einen enthielt er inhaltliche Fehler (etwa zu Kontraindikation, Anwendung während der Stillzeit und Dauer der Wiederherstellung der Fruchtbarkeit nach der Anwendung) und zum anderen keinen einzigen Literaturverweis, um seine Aussagen zu belegen. Mehrere Wikipedia-Einträge erreichten jedoch sehr hohe Korrektheitswerte von 4,67 – die Einträge zur Antibabypille, zur Pille für den Mann, zur Pille danach, zur Sterilisation des Mannes und zum Zykluscomputer. Dass hier nicht der Maximalwert von 5,0 erreicht wurde, lag *nicht* daran, dass falsche Informationen vorhanden waren, sondern an vereinzelt fehlenden Quellennachweisen. Im Durchschnitt erhielten die 25 Artikel einen Korrektheitsscore mit einem Mittelwert von M = 3,85, was angesichts eines Maximalwertes von 5 ausdrückt, dass ein Großteil der Artikel einen hohen Korrektheitsgrad aufwies (Tab. 3).

**Table 3:** 

Verhütungsmethode	Korrektheit	Vollständigkeit	Neutralität	Verständlichkeit	Aktualität	Gesamtbewertung	Modified-DISCERN-Score
1. Antibabypille	4,67	4,00	3,83	3,00	4,00	3,33	40,00
2. Coitus interruptus	3,33	4,50	5,00	1,00	5,00	4,00	39,00
3. Diaphragma	3,67	2,17	4,00	5,00	2,00	2,00	32,33
4. Dreimonatsspritze	1,67	2,33	4,00	3,00	4,00	2,00	26,67
5. Etonogestrel-Implantat	3,33	3,50	3,00	2,00	1,00	2,67	28,67
6. Femidom	4,00	2,00	3,50	5,00	2,00	2,00	32,00
7. Hormonpflaster	2,33	2,00	3,50	3,00	4,00	1,00	26,00
8. Hormonspirale	4,00	2,50	2,50	3,00	2,00	2,00	29,00
**9. Intrauterinpessar**	**4,33**	**4,00**	**5,00**	**3,00**	**4,00**	**4,00**	**42,00**
10. Knaus-Ogino-Methode	4,22	3,83	3,83	2,33	4,00	3,00	37,00
**11. Kondom**	**4,00**	**4,00**	**4,17**	**5,00**	**4,00**	**3,00**	**40,33**
12. Kupferkette	3,78	3,00	3,83	3,00	1,00	2,67	31,33
13. LAM-Methode	4,44	3,33	3,00	2,00	2,00	2,67	33,00
14. Minipille	3,00	2,83	4,33	1,00	2,00	2,67	28,33
15. Pille danach	4,67	2,50	3,50	1,00	2,00	3,00	32,00
16. Pille für den Mann	4,67	2,33	3,83	1,00	4,00	3,00	33,00
17. Portiokappe	1,89	1,83	4,00	5,00	2,00	2,00	26,33
18. Spermizid	3,56	1,33	3,50	1,00	2,00	1,67	24,67
19. Sterilisation der Frau	4,56	1,33	2,83	1,00	4,00	2,00	29,67
**20. Sterilisation des Mannes**	**4,67**	**4,00**	**4,50**	**5,00**	**4,00**	**4,67**	**44,33**
21. Symptothermale Methode	3,89	4,33	4,67	5,00	2,00	4,00	40,00
22. Temperaturmethode	4,22	2,83	3,50	5,00	2,00	2,00	34,33
23. Verhütungsring	4,22	3,00	4,67	4,00	3,00	3,00	38,00
24. Verhütungsschwamm	4,33	3,33	3,50	3,00	2,00	3,00	34,33
25. Zykluscomputer	4,67	1,00	3,67	1,00	2,00	1,67	28,00
**Mittelwert M (insgesamt)**	**3,85** (SD = 0,85)	**2,87** (SD = 1,00)	**3,83** (SD = 0,64)	**2,93** (SD = 1,57)	**2,80** (SD = 1,16)	**2,68** (SD = 0,87)	**33,21** (SD = 5,56)

Darstellung der Qualitätsdimensionen auf der Basis des Messinstruments in Tab. 2 anhand von Mittelwerten (jeweils 3 Codierende und 1–3 Items). Einschätzung der modified DISCERN-Items auf 5‑stufigen Ratingskalen, wobei 1 Punkt für sehr geringe Qualität und 5 Punkte für sehr hohe Qualität stehen. Der DISCERN-Score besteht aus der Summe der 10 DISCERN-Items. Da in dieser Tabelle die 10 DISCERN-Items (siehe Tab. 2) nicht einzeln, sondern nach Qualitätsdimensionen zusammengefasst dargestellt sind, addieren sich die dargestellten Werte nicht direkt zu dem DISCERN-Score. Die 3 Artikel mit der höchsten Qualität laut Modified-DISCERN-Score (> 40,00) sind durch Fettdruck hervorgehoben. Die Mittelwerte M (insgesamt) basieren auf den auf 2 Nachkommastellen gerundeten Einzelwerten

*LAM-Methode* Laktationsamenorrhö-Methode, Methode der natürlichen Empfängnisverhütung durch Stillen

### Vollständigkeit der Verhütungsinformationen

Neben der inhaltlichen Korrektheit spielt die Vollständigkeit als Qualitätskriterium eine zentrale Rolle. Denn ein Wikipedia-Artikel, der ausschließlich korrekte und gut belegte Informationen bietet, kann trotzdem geringe Informationsqualität aufweisen, wenn viele wichtige Informationen fehlen. Die Vollständigkeit wurde gemäß unserem auf dem Modified-DISCERN-Index basierenden Messinstrument (Tab. 2) mit 2 Kriterien gemessen: Zum einen wurde erfasst, ob der Wikipedia-Artikel alle sieben Hauptthemen zur jeweiligen Verhütungsmethode enthält – also 1. Wirkungsmechanismus, 2. Sicherheit, 3. Anwendung, 4. Vorteile, 5. Nachteile, 6. Kosten und 7. Gegenanzeige behandelt. Zum anderen wurde erfasst, ob für jedes dieser sieben Hauptthemen auch alle für die jeweilige Methode spezifischen relevanten Unterthemen dargestellt werden. So hat etwa das Kondom andere Vorteile, indem es z. B. auch vor sexuell übertragbaren Infektionen schützt, als die Pille, die z. B. Beschwerden bei Endometriose verringern kann. Beide Vollständigkeitsaspekte wurden zu einem Vollständigkeitswert kombiniert.

Hinsichtlich der Vollständigkeit wiesen einige Einträge große Defizite auf und erreichten Scores im unteren Bereich zwischen 1 und 2 (Tab. 3). Schlusslicht war der rudimentäre Eintrag zum Zykluscomputer (Vollständigkeitswert von 1,00), er bot nur einen unzureichenden Überblick über die verschiedenen Arten von Zykluscomputern und erwähnte keinerlei Vor- und Nachteile sowie Kosten. Dagegen konnte dem Wikipedia-Eintrag zum Coitus interruptus mit einem Score von 4,50 eine sehr hohe Vollständigkeit bescheinigt werden. Hier fehlte lediglich ein Anwendungshinweis zum Vorgehen, wenn bereits kürzlich vor dem Geschlechtsverkehr ejakuliert wurde. Insgesamt erwies sich die Vollständigkeit der untersuchten Wikipedia-Artikel als mittelmäßig (M = 2,87).

### Neutralität der Verhütungsinformationen

Die Wikipedia als Online-Enzyklopädie ist einem neutralen Standpunkt verpflichtet [14]. Das bedeutet, dass Wikipedia-Einträge nicht dazu dienen dürfen, Werbung für ein Verhütungsprodukt zu machen. Ebenso dürfen Wikipedia-Artikel nicht durch weltanschauliche oder religiöse Vorbehalte gegenüber Verhütungsmethoden geprägt sein, sondern müssen den aktuellen Forschungsstand ausgewogen präsentieren. Neutralität wurde anhand von 2 Variablen operationalisiert (Tab. 2): zum einen anhand der Anzahl polarisierender Behauptungen ohne Quellennachweise und zum anderen anhand der Ausgewogenheit der Darstellung der tatsächlichen Vor- und Nachteile einer Methode (Tab. 2).

Insgesamt wurden hinsichtlich Neutralität eher wenige Qualitätsprobleme identifiziert (M = 3,83; Tab. 3). Die Wikipedia-Einträge zum Intrauterinpessar und zum Coitus interruptus erhielten jeweils die Höchstbewertung von 5,00 Punkten für Neutralität, da die Vor- und Nachteile der Methoden ausgewogen dargestellt waren. Die Hormonspirale dagegen wurde bei einem Score von 2,50 als eher wenig neutral eingestuft, da hier die Nachteile der Verhütungsmethode sehr umfangreich und detailliert dargestellt, Vorteile hingegen nicht bzw. nur indirekt genannt wurden. Zudem wurde eine polarisierende Aussage identifiziert („Die angeblich ‚lokale‘ Wirkung ist nicht möglich, nicht wissenschaftlich nachzuweisen, und selbst der Hersteller gab auf Nachfrage zu, dass sie nicht erwiesen sei.“), bei der als Quelle nur ein Transkript eines Radiobeitrags auf der Website „www.risiko-hormonspirale“ hinterlegt war.

### Verständlichkeit der Verhütungsinformationen

Gesundheitsinformationen im Allgemeinen und Verhütungsinformationen im Besonderen können abstrakt und komplex sein (z. B. hormonelles Geschehen während des Menstruationszyklus). Daher ist es für eine qualitätsvolle Verhütungskommunikation wichtig, auch die Verständlichkeit zu sichern. Eine wichtige Methode ist dabei die Visualisierung durch Fotos, Zeichnungen oder Videos. Dementsprechend ist das Anbieten sinnvoller Visualisierungen ein zentrales Qualitätskriterium von Gesundheitsinformationen gemäß dem Modified-DISCERN-Index (Tab. 2). Während der Wikipedia-Beitrag zur Sterilisation des Mannes durch Visualisierungen die Methode gut veranschaulichte und dafür einen Maximalwert von 5,00 erhielt, bekam der Eintrag zur Sterilisation der Frau lediglich einen Score von 1,00, da diese Methode (die nur als Unterpunkt des allgemeinen Sterilisationsartikels behandelt wird) keine einzige Veranschaulichung bot. Insgesamt war die Verständlichkeit der *N* = 25 Artikel in der deutschsprachigen Wikipedia gemessen an den bereitgestellten Visualisierungen mit einem Mittelwert von M = 2,93 mittelmäßig. Zuweilen enthielten Beiträge zwar Visualisierungen, diese waren aber nicht genau erklärt und/oder zum Verständnis der Verhütungsmethode nicht relevant (z. B. wird im deutschsprachigen Wikipedia-Artikel zum Etonogestrel-Implantat nicht das Implantat selbst, sondern nur der Applikator gezeigt und zwar ohne jede erläuternde Erklärung).

Neben der Verständlichkeit durch Visualisierungen ist auch die sprachliche Verständlichkeit ein Qualitätskriterium. Sie ist kein Bestandteil des Modified-DISCERN-Index und wird in Qualitätsstudien typischerweise ergänzend mit dem *Flesch-Reading-Ease-Index* erfasst [38–40]. Gemäß diesem etablierten Messinstrument mit dem Wertebereich von 0 (sehr schwere Verständlichkeit) bis 100 (sehr leichte Lesbarkeit und Verständlichkeit) ist die textliche Verständlichkeit umso größer, je kürzer die verwendeten Sätze und Wörter sind (Tab. 2). Die untersuchten Artikel erreichten textuelle Verständlichkeitswerte gemäß Flesch-Reading-Ease-Index zwischen 28 und 51 (M = 37,16, SD = 8,64), was als schwere Lesbarkeit gilt.

### Aktualität der Verhütungsinformationen

Im Feld der Verhütungsmethoden gibt es kontinuierlich Veränderungen im Entwicklungs- und Forschungsstand. Unser Messinstrument enthält daher die Aktualität der Informationen als wichtiges Qualitätskriterium (Tab. 2). Als Indikatoren für fehlende Aktualität galten das längere Zurückliegen der letzten Bearbeitung und das Vorhandensein toter Verlinkungen.

Am wenigsten aktuell waren die Artikel zum Hormonimplantat (Etonogestrel-Implantat) und zur Kupferkette mit einem Minimalrating von 1,00 (Tab. 3). Beide wurden inhaltlich und formal im vergangenen Jahr gar nicht bearbeitet. Zudem wies der Eintrag zum Hormonimplantat 6 tote Verlinkungen auf. Dagegen hob sich der Artikel zum Coitus interruptus als der aktuellste ab mit einem Maximalwert von 5,00: Er wurde in den vergangenen 12 Monaten inhaltlich und formal bearbeitet und enthielt keine toten Links. Im Durchschnitt erreichten die 25 Wikipedia-Einträge zu Verhütungsmethoden eine mittlere Aktualität (M = 2,80).

### Gesamtqualität der Verhütungsinformationen

**Figure 1:**
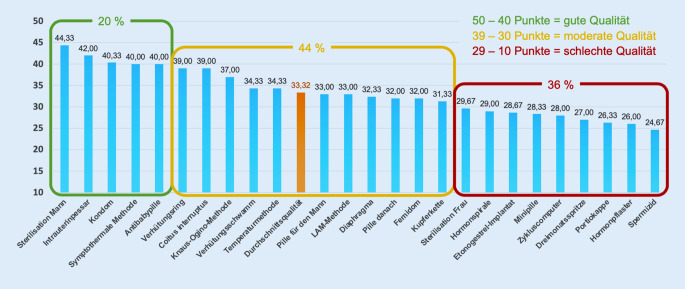

Die Gesamtbilanz für die fünf zentralen Qualitätsdimensionen stellt sich so dar, dass 2 Kriterien (Korrektheit und Neutralität) mit Mittelwerten von rund 4 eher gut, 3 Kriterien (Vollständigkeit, Verständlichkeit und Aktualität) jedoch mit Mittelwerten um 3 eher mittelmäßig abschnitten (Tab. 3). Dies beeinflusste auch den Gesamteindruck: Die 3 geschulten Codierenden verliehen den Artikeln im Ganzen einen Qualitätsscore von 2,68. Fasst man zudem rechnerisch alle 10 Modified-DISCERN-Items zu einem Summenscore zusammen (Tab. 2), so ergibt sich ein Modified-DISCERN-Score von 33,21, was nach der üblichen DISCERN-Klassifikation [36] moderate Qualität anzeigt. In der Gesamtschau zeigt sich, dass die 25 Artikel in 3 Qualitätsgruppen fallen, wobei die größte Gruppe (44 %) aus Artikeln moderater Qualität besteht (Abb. 1).

Als beste Artikel in Hinblick auf die Gesamtqualität erwiesen sich jene zur Sterilisation des Mannes mit einem Modified-DISCERN-Score von 44,33, zum Intrauterinpessar (42,00) und zum Kondom (40,33). Diese Artikel zeichneten sich dadurch aus, dass sie auf nahezu allen Qualitätsdimensionen Werte zwischen 4 und 5 erreichten. Der Beitrag zur Sterilisation des Mannes hob sich am stärksten dadurch positiv ab, dass einschlägiges Bildmaterial die Verständlichkeit des Artikels erhöhte. Eine bessere Gesamtqualität gemäß Modified-DISCERN-Score stand in engem positiven Zusammenhang mit der Anzahl der Überarbeitungen (r = 0,52, *p* = 0,008) und der Anzahl der Autor*innen (r = 0,50, *p* = 0,010).

## Diskussion

### Interpretation der Befunde zu den sechs Forschungsfragen

Hinsichtlich Korrektheit (F1) schnitten die deutschsprachigen Wikipedia-Artikel zum Thema Empfängnisverhütung insgesamt gut ab: Fehlinformationen kamen kaum vor, aber Belege durch Quellen fehlten häufiger.

Hinsichtlich Vollständigkeit (F2) zeigte sich nur eine moderate Qualität. Hier wurden zuweilen Hauptthemen (z. B. Anwendung, Kosten oder Gegenanzeige einer Methode) gar nicht erwähnt. Ebenso waren Informationen zu Unterthemen (z. B. Auflistung der einzelnen Vor- und Nachteile einer Methode) häufig unvollständig. Dass manche Artikel bei der Vollständigkeit so schlecht abschnitten, lag auch an ihrer Kürze. Zwischen der Artikellänge und der Vollständigkeit der präsentierten Information bestand jedenfalls eine enge positive Korrelation (r = 0,68, *p* < 0,001).

Hinsichtlich Neutralität (F3) zeigte sich insgesamt eine gute Qualität: Offensichtlich tendenziös oder ideologisch verzerrt waren die Beiträge in der Regel nicht. Unausgewogenheit konnte aber dadurch entstehen, dass der Darstellung von Vor- und Nachteilen einer Verhütungsmethode unterschiedliches Gewicht beigemessen wurde. So nehmen Nachteile der Antibabypille oder der Hormonspirale vergleichsweise großen Raum im jeweiligen Wikipedia-Artikel ein im Vergleich zu den evidenzbasierten Vorteilen.

Bei der Verständlichkeit (F4) erreichten die Wikipedia-Beiträge nur moderate Qualität, da sie wenige und teilweise auch kaum hilfreiche Visualisierungen anboten. Dabei ist es die große Chance einer Online-Enzyklopädie, dass sie im Unterschied zum Printlexikon ohne Platzbeschränkungen und Zusatzkosten Zeichnungen, Fotos und sogar Videos bereitstellen kann. Im Unterschied zur englischsprachigen Wikipedia, die von diesen Optionen häufiger Gebrauch macht und z. B. in ihrem Artikel zum Hormonimplantat^9^ in jeweils einem Video zeigt, wie das Implantat eingesetzt und entfernt wird, enthielt kein einziger der untersuchten deutschsprachigen Artikel ein Video. Hinsichtlich Textverständlichkeit steht die Wikipedia vor einem gewissen Dilemma: Denn einerseits sind längere Texte im Sinne der Vollständigkeit der Informationen wünschenswert. Andererseits sind längere und komplexere Texte für Zielgruppen mit geringerer formaler Bildung schwerer verständlich. Die Lesbarkeitsindizes fielen für die untersuchten Artikel aufgrund langer Sätze und Wörter moderat aus.

Mängel bei der Aktualität (F5), etwa seltene Überarbeitungen und viele tote Links, erwiesen sich bei einigen Verhütungsartikeln als Qualitätsproblem.

Insgesamt bleibt festzuhalten, dass von den fünf Qualitätskriterien zwei (Korrektheit, Neutralität) gut und drei (Vollständigkeit, Verständlichkeit, Aktualität) mittelmäßig ausfielen, was zu einer insgesamt moderaten Gesamtqualität (F6) führte, die auf Verbesserungspotenziale hindeutet. Die moderate Gesamtqualität und die schwere Lesbarkeit der Wikipedia-Artikel zum Thema Empfängnis- bzw. Zeugungsverhütung entsprechen den Befunden früherer Wikipedia-Studien zu anderen gesundheitsbezogenen Themen (Tab. 4).

**Table 4:** 

Autor*innen	Thema	Modified-DISCERN-Score (Mittelwert/*Median)	Modified-DISCERN-Score (Range)	Flesch-Reading-Ease-Index (Mittelwert)	Flesch-Kincaid-Grade Level-Index (Mittelwert)
Azer (2015; [37])	Atemwegserkrankungen	26,40	14,67–38,33	–	15
Azer et al. (2015; [36])	Kardiovaskuläre Krankheiten	33,00*	28,00–45,00	–	14
**Vorliegende Studie**	**Verhütung**	**33,21**	**24,67–44,33**	**37**	**–**
Azer (2014; [35])	Gastroenterologie und Hepatologie	34,31	15,60–43,60	26	–
Handler et al. (2021; [38])	Beckenbodenerkrankungen	34,43	21,00–43,00	33	–
Simpson et al. (2018; [39])	Gehörverlust	35,63	25,00–44,00	–	13
Suwannakhan et al. (2020; [40])	Anatomie	36,10	20,00–45,00	43	–

*Modified-DISCERN-Score* von 10 (niedrigste Qualität) bis 50 (höchste Qualität): 10–29 = schlechte Qualität; 30–39 = moderate Qualität; 40–50 = gute Qualität. *Flesch-Reading-Ease-Index* von 0 (schwerste Lesbarkeit) bis 100 (leichteste Lesbarkeit): 0–30 = sehr schwer lesbar; 31–50 = schwer lesbar; 51–60 = mittelschwer lesbar; 61–79 = mittel lesbar; 71–80 = mittelleicht lesbar; 81–90 = leicht lesbar; 91–100 = sehr leicht lesbar. Der *Flesch-Kincaid-Grade-Level-Index* ist eine Weiterentwicklung des Flesch-Reading-Ease-Index, wobei die *Grade Level* den Jahrgangsstufen im amerikanischen Bildungssystems entsprechen. Beide Flesch-Indizes nutzen die gleichen Kernmaße (Wort- und Satzlänge) der Lesbarkeit, aber mit unterschiedlicher Gewichtung. Die präsentierten Flesch-Kincaid-Werte von 13–15 entsprechen dem Niveau von Hochschulabsolvent*innen (sehr schwere Lesbarkeit)

### Limitationen und Stärken der Studie

Das detaillierte Messinstrument ist gleichermaßen eine Stärke und Schwäche der vorliegenden Arbeit. Denn alle darin enthaltenen Konkretisierungen laden natürlich vielfache Kritik ein, da jede Detailentscheidung auch anders getroffen werden könnte. Wir sind jedoch überzeugt, dass die Formalisierung Vorteile gegenüber den sonst üblichen intransparenten subjektiven Expertenurteilen bietet. Denn die Detailmessungen erlauben bessere Vergleichsmöglichkeiten und können auch Anregungen für weitere Instrumententwicklungen liefern. Die Studie beschränkt sich auf Vergleiche innerhalb der deutschsprachigen Wikipedia. Durch Anpassungen des Instruments ist es zukünftig möglich, Verhütungsinformationen auch auf weiteren Online- und Social-Media-Plattformen zu untersuchen (z. B. englischsprachige Wikipedia, Websites von Fachorganisationen, YouTube). Abschließend sei noch auf eine generelle Limitation der Social-Media-Forschung verwiesen: Angesichts der Schnelllebigkeit der Plattformen bietet jedes Studienergebnis immer nur eine flüchtige Momentaufnahme. Im konkreten Untersuchungsfall wäre es sogar wünschenswert, wenn die hier aufgezeigten Qualitätsmängel schon bald gar nicht mehr vorhanden wären.

### Fazit und Ausblick

Die vorliegende Studie hat erstmals objektiv nachvollziehbare Daten zur Qualität von Verhütungsinformationen in der deutschsprachigen Wikipedia präsentiert und dabei große Qualitätsdifferenzen zwischen den 25 Artikeln zu verschiedenen Methoden der Empfängnis- bzw. Zeugungsverhütung aufgezeigt. Die Befunde sowie das bereitgestellte Instrument (https://osf.io/rebqh/) können genutzt werden, um in Zukunft Verhütungsinformationen auf weiteren Online- und Social-Media-Plattformen zu analysieren und zu vergleichen. Weiterhin können die vorliegenden Befunde in der Praxis fruchtbar gemacht werden, indem die nachgewiesenen Mängel in der Inhaltsqualität (z. B. geringe Aktualität vieler Einträge) durch eine zielgerichtete Bearbeitung der Wikipedia-Einträge behoben werden (z. B. strukturiertes Einpflegen relevanter aktueller Fachpublikationen; Aktualisierung von toten Links; Integration von mehr aussagekräftigen Visualisierungen). Darüber hinaus gilt es zu überlegen, wie strukturell vorzugehen ist, damit Fachexpertise zur sexuellen und reproduktiven Gesundheit sowie zu weiteren Gesundheitsthemen in Zukunft noch schneller und umfassender in die Wikipedia einfließen kann, etwa durch Kooperationsprojekte zwischen der Wikipedia-Community, der Wikimedia Foundation und wissenschaftlichen Fachgesellschaften oder Fachorganisationen.

## Footnotes

1 Der vorliegende Beitrag fokussiert auf die Verhütung von Empfängnis/Zeugung. Die Verhütung von sexuell übertragbaren Infektionen (STI) wird nicht behandelt.

2 https://de.wikipedia.org/wiki/Kondom (letzter Zugriff: 12.04.2022).

3 Fachlich geprüfte deutschsprachige Verhütungsinformationen werden im Internet unter anderem bereitgestellt vom Berufsverband der Frauenärzte e. V. (https://www.frauenaerzte-im-netz.de/familienplanung-verhuetung/), von pro familia Deutschland (https://www.profamilia.de/themen/verhuetung/) und von der BZgA (www.loveline.de; www.familienplanung.de; letzter Zugriff: 12.04.2022).

4 BZgA-interne Seitenabrufstatistik.

5 https://www.familienplanung.de/verhuetung/verhuetungsmethoden/kondom-fuer-ihn-sie/kondom/ (letzter Zugriff: 12.04.2022).

6 Der Modified-DISCERN-Index stellt eine Weiterentwicklung des ursprünglichen DISCERN-Index ([34]; http://www.discern.org.uk/) dar: Er bezieht sich speziell auf Online-Informationen und berücksichtigt auch deren inhaltliche Korrektheit.

7 In der Fachliteratur wird oft mit Expertenurteilen gearbeitet, um die Gesamtqualität oder einzelne Qualitätsdimensionen von Online-Gesundheitsinformationen einzuschätzen. So kann z. B. eine Fachkraft den Wikipedia-Eintrag zur Antibabypille durchlesen und anschließend gemäß Modified-DISCERN-Index [35, 36] die Vollständigkeit der Informationen auf einer fünfstufigen Ratingskala beurteilen von 1 (sehr geringe Vollständigkeit) bis 5 (sehr hohe Vollständigkeit). Dieses in der Fachliteratur oft angewendete Vorgehen hat den Vorteil, dass es ohne großen Aufwand spontan umzusetzen ist, sofern entsprechende Fachkräfte mitarbeiten. Nachteilig ist jedoch, dass unklar bleibt, aufgrund welcher Überlegungen und Eindrücke die subjektive Skalenbewertung im Einzelnen erfolgt. Unser Instrument konkretisiert die Regeln für die Codierung.

8 Eine Normseite ist definiert über 1800 Zeichen inklusive Leerzeichen.

9 https://en.wikipedia.org/wiki/Contraceptive_implant (letzter Zugriff: 12.04.2022).
